# Supplementary material for: Investigating the moments of “aha” and “hmm” through acoustic analysis of voice and speech in pre-service physics teacher education–A novel method for identifying significant learning moments
Source: PLoS One. 2025 Jan 24;20(1):e0314344. doi: 10.1371/journal.pone.0314344 (PMC11761114; doi:10.1371/journal.pone.0314344)
Supplement: Attachment — Submitted filename: Response to reviewers.docx [file pone.0314344.s002.docx]

PONE-D-23-20273
Acoustic characteristics of voice and speech in learning dialogues
PLOS ONE

Additional Editor Comments:

Dear Author,
Thank you for drafting a paper on an interesting topic to analyze acoustic features of voice recordings. However, there are certain major concerns here. To begin with, the paper has serious methodological concerns regarding its sample size, which is too small to arrive at generalized outcomes, and data collection process. Face masks during recording can affect participants' voice quality, which will further impact analyzing the acoustic features of collected data. The abstract seems like a direct pickup of results along with acronyms without any full form. It should be precise about the research objectives, methods, and results written in a manner of providing a general description including these. Overall, the paper requires a major revision - a fresh new start which will also affect the outcomes and give it better clarity - on that part since its entirety including results is based on that. Therefore, the paper in its current version is not ready for publication in this journal. But it would be great to work on those aspects along with those pointed out by reviewers to rewrite the paper and make a fresh submission to this or any other journal of repute. The topic definitely has relevance and hence rework on the paper is recommended. Thank you.

**Reply:** Thank you for noticing the relevance of the topic. We have now rewritten the article and strengthened it. Introduction has now a stronger link to previous research. For generalizing the results, generalized mixed model analyses were conducted as suggested by one reviewer. For face masks we cannot do anything about since the data was collected during Covid-19 pandemic. But the effect of face masks has been discussed. The masks should not affect the results as participants wore masks throughout the data collection and the comparisons in the changes of the acoustic parameters were done by comparing individual changes. Then, the effect of a face mask is the same in each category to be studied here. We have not compared the numerical spectral characteristics to previously suggested values indicating certain voice qualities or phonation types. That is to say, we don´t propose certain values to be indicating the speech categories, we suggest that the changes make the distinctions.

Reviewers' comments:

Reviewer's Responses to Questions

**Comments to the Author**

1. Is the manuscript technically sound, and do the data support the conclusions?

The manuscript must describe a technically sound piece of scientific research with data that supports the conclusions. Experiments must have been conducted rigorously, with appropriate controls, replication, and sample sizes. The conclusions must be drawn appropriately based on the data presented.

Reviewer #1: No

Reviewer #2: Yes

2. Has the statistical analysis been performed appropriately and rigorously?

Reviewer #1: No

Reviewer #2: Yes

3. Have the authors made all data underlying the findings in their manuscript fully available?

The [PLOS Data policy](https://eur03.safelinks.protection.outlook.com/?url=http%3A%2F%2Fwww.plosone.org%2Fstatic%2Fpolicies.action%23sharing&data=05%7C01%7Ckati.h.jarvinen%40jyu.fi%7C69eebd7d82f74e723a7308dbbf538a31%7Ce9662d58caa44bc1b138c8b1acab5a11%7C1%7C0%7C638314137744248314%7CUnknown%7CTWFpbGZsb3d8eyJWIjoiMC4wLjAwMDAiLCJQIjoiV2luMzIiLCJBTiI6Ik1haWwiLCJXVCI6Mn0%3D%7C3000%7C%7C%7C&sdata=GFPIkL9K0LdqqGcAtOLwSEbbk284FOXaZPMD4DBQZMk%3D&reserved=0) requires authors to make all data underlying the findings described in their manuscript fully available without restriction, with rare exception (please refer to the Data Availability Statement in the manuscript PDF file). The data should be provided as part of the manuscript or its supporting information, or deposited to a public repository. For example, in addition to summary statistics, the data points behind means, medians and variance measures should be available. If there are restrictions on publicly sharing data—e.g. participant privacy or use of data from a third party—those must be specified.

Reviewer #1: Yes

Reviewer #2: Yes

4. Is the manuscript presented in an intelligible fashion and written in standard English?

PLOS ONE does not copyedit accepted manuscripts, so the language in submitted articles must be clear, correct, and unambiguous. Any typographical or grammatical errors should be corrected at revision, so please note any specific errors here.

Reviewer #1: Yes

Reviewer #2: Yes

5. Review Comments to the Author

Please use the space provided to explain your answers to the questions above. You may also include additional comments for the author, including concerns about dual publication, research ethics, or publication ethics. (Please upload your review as an attachment if it exceeds 20,000 characters)

Reviewer #1: I have carefully read the paper, including the attached responses to a previous version of paper.

While the paper asks some currently unanswered research questions, relating to how different kinds of speech types within a learning scenario may be marked in a person's voice, I unfortunately do not think that this paper currently lives up to the quality of research published in Plos ONE. While some aspects highlighted below can be improved (e.g. the nature of the statistical analysis), other aspects cannot be remedied without additional data collection.
**Reply:** We thank you for taking the time to review our study and for the valuable comments. We have addressed these as follows:
Major issues:
1. Sample size: A key issue that cannot be readily addressed is that the sample size is very small - only 5 pairs of students were recorded, with one student dropping out, leaving a final sample size on 9. This small sample severely limits the generalisability of the findings.
**Reply:** We find that the sample size of speech samples was quite sufficient however spoken by only nine people. We have addressed the generalizability in next reply.

2. Analysis: Given the very small number of participants, data should have been analysed using linear mixed models to account for the effects of individual speakers and the effect of individual stimuli. Since this was not done, it can currently not be ruled out that any reported effects are driven by individual speakers.
**Reply:** Thank you for pointing this out. We have added generalized linear mixed model to the statistical analysis. According to the results, no individual effect was found. Also, the effect sizes confirm the results to be solid.

3. Procedure: The participants were furthermore wearing masks, as a previous reviewer has pointed out. While this was perhaps unavoidable at the time, mask wearing has further implications for generalisability as it does affect how people communicate with one another.
**Reply:** Thank you for addressing this. Wearing a mask can affect the communication. However, people had been quite used to wearing masks at the time of data collection. We find that masks do not affect the outcome of this study, since they were worn throughout the research and the effect should be the same in every learning session as well between the speech categories.

4. Selection of speech utterances: Utterances in the different conditions (understanding, confusion, etc.) were select by subjective evaluations of two experimenters. While the two experimenters agreed with one another, it is methodologically not ideal to have experimenters impose a “ground truth” onto these recording. There is a difference between experimenters perceiving different “speech types” and speakers truly expressing confusion/understanding. While there is likely some degree of overlap between the subjective judgements and the actual “ground truth”, it would have been nice to see more efforts being made to identify true instances of “confusion”, etc.
For example, considering the small number of participants, I assume, it would have been feasible to ask participants to review their conversations and themselves mark out instances of “understanding”, “confusion”, “explaining”.

**Reply:** We acknowledge this comment, but we would like to point that the selection of speech samples was made from the whole recordings and they could be identified by the context. Had we asked the participants to mark these moments themselves, it would/could have most likely affected the spontaneity of the learning discussions as the participants´ attention would have been on the instances rather than the tasks.

Reviewer #2: I am pleased to comment that this paper represents a technically sound piece of scientific research, characterized by a well-structured methodology, robust data analysis, and conclusions that are effectively supported by the presented data. The methodology section is comprehensive and provides a clear and detailed description of the experimental procedures. This paper makes a valuable contribution to the field and has the potential to advance our understanding of learning dialogues. I recommend acceptance of this manuscript for publication in the journal.

**Reply:** Thank you for taking the time to review our study. We are thankful for these comments.

**Do you want your identity to be public for this peer review?** For information about this choice, including consent withdrawal, please see our [Privacy Policy](https://eur03.safelinks.protection.outlook.com/?url=https%3A%2F%2Fwww.plos.org%2Fprivacy-policy&data=05%7C01%7Ckati.h.jarvinen%40jyu.fi%7C69eebd7d82f74e723a7308dbbf538a31%7Ce9662d58caa44bc1b138c8b1acab5a11%7C1%7C0%7C638314137744248314%7CUnknown%7CTWFpbGZsb3d8eyJWIjoiMC4wLjAwMDAiLCJQIjoiV2luMzIiLCJBTiI6Ik1haWwiLCJXVCI6Mn0%3D%7C3000%7C%7C%7C&sdata=Sgm%2FhQlsQnORqemN8BBIw5jYKoreqaLkEtOfu71dWzY%3D&reserved=0).

Reviewer #1: No

Reviewer #2: **Yes:** INA SURYANI
