# Supplementary material for: Investigating the moments of “aha” and “hmm” through acoustic analysis of voice and speech in pre-service physics teacher education–A novel method for identifying significant learning moments
Source: PLoS One. 2025 Jan 24;20(1):e0314344. doi: 10.1371/journal.pone.0314344 (PMC11761114; doi:10.1371/journal.pone.0314344)
Supplement: Attachment — Submitted filename: Response to reviewers.docx [file pone.0314344.s003.docx]

Academic editor´s comments:

Journal Requirements:

When submitting your revision, we need you to address these additional requirements.

1. Please ensure that your manuscript meets PLOS ONE's style requirements, including those for file naming. The PLOS ONE style templates can be found at
   <https://journals.plos.org/plosone/s/file?id=wjVg/PLOSOne_formatting_sample_main_body.pdf> and  
   <https://journals.plos.org/plosone/s/file?id=ba62/PLOSOne_formatting_sample_title_authors_affiliations.pdf>

**Response**: We have ensured this.

2. We note that you have referenced (Unpublished master thesis) which has currently not yet been accepted for publication. Please remove this from your References and amend this to state in the body of your manuscript: (ie “Bewick et al. [Unpublished]”) as detailed online in our guide for authors 
<http://journals.plos.org/plosone/s/submission-guidelines#loc-reference-style>

**Response**: This thesis was necessary to keep, since it validates VHI for Finnish speakers, therefore we moved it to Supporting information (page 32). And amended this to state in the manuscript, line 193.

3. Please review your reference list to ensure that it is complete and correct. If you have cited papers that have been retracted, please include the rationale for doing so in the manuscript text, or remove these references and replace them with relevant current references. Any changes to the reference list should be mentioned in the rebuttal letter that accompanies your revised manuscript. If you need to cite a retracted article, indicate the article’s retracted status in the References list and also include a citation and full reference for the retraction notice.

**Response**: References have been reviewed and corrected.

Additional Editor Comments: 
 
Thank for making the revisions. Please further improve the manuscript by considering the reviewers' comments. 
 
While revising your submission, please upload your figure files to the Preflight Analysis and Conversion Engine (PACE) digital diagnostic tool, [https://pacev2.apexcovantage.com/](https://eur03.safelinks.protection.outlook.com/?url=https%3A%2F%2Fc05y1x9s.r.us-east-2.awstrack.me%2FL0%2Fhttps%3A%252F%252Fpacev2.apexcovantage.com%252F%2F1%2F010f0191948db704-7c83b35d-16b5-454c-8766-c1d23c62641e-000000%2FTV-XAM8mB-WOQl_POIqmQ6_fZkQ%3D174&data=05%7C02%7Ckathanja%40jyu.mail.onmicrosoft.com%7C7a07683149de4d13737608dcc6b0d93a%7Ce9662d58caa44bc1b138c8b1acab5a11%7C1%7C0%7C638603711603577685%7CUnknown%7CTWFpbGZsb3d8eyJWIjoiMC4wLjAwMDAiLCJQIjoiV2luMzIiLCJBTiI6Ik1haWwiLCJXVCI6Mn0%3D%7C0%7C%7C%7C&sdata=QoN34OH72QLnKO6OPClyF6p3fr6%2Fmn2u3pRBVbxoH%2Bo%3D&reserved=0). PACE helps ensure that figures meet PLOS requirements. To use PACE, you must first register as a user. Registration is free. Then, login and navigate to the UPLOAD tab, where you will find detailed instructions on how to use the tool. If you encounter any issues or have any questions when using PACE, please email PLOS at [figures@plos.org](mailto:figures@plos.org). Please note that Supporting Information files do not need this step.

**Response**: The figures have been uploaded to PACE and ensured they meet the requirements. Figure 7 was remade in color for clearer distinctions.

Reviewers´ comments:

Reviewer's Responses to Questions

**Comments to the Author** 
 
1. Is the manuscript technically sound, and do the data support the conclusions? 
 
The manuscript must describe a technically sound piece of scientific research with data that supports the conclusions. Experiments must have been conducted rigorously, with appropriate controls, replication, and sample sizes. The conclusions must be drawn appropriately based on the data presented.

Reviewer #1: Yes

Reviewer #2: Yes

2. Has the statistical analysis been performed appropriately and rigorously?

Reviewer #1: Yes

Reviewer #2: Yes

3. Have the authors made all data underlying the findings in their manuscript fully available? 
 
The [PLOS Data policy](https://eur03.safelinks.protection.outlook.com/?url=http%3A%2F%2Fc05y1x9s.r.us-east-2.awstrack.me%2FL0%2Fhttp%3A%252F%252Fwww.plosone.org%252Fstatic%252Fpolicies.action%2523sharing%2F1%2F010f0191948db704-7c83b35d-16b5-454c-8766-c1d23c62641e-000000%2F1NloED7OszUU2Bsz6gpoY3R9lWg%3D174&data=05%7C02%7Ckathanja%40jyu.mail.onmicrosoft.com%7C7a07683149de4d13737608dcc6b0d93a%7Ce9662d58caa44bc1b138c8b1acab5a11%7C1%7C0%7C638603711603559327%7CUnknown%7CTWFpbGZsb3d8eyJWIjoiMC4wLjAwMDAiLCJQIjoiV2luMzIiLCJBTiI6Ik1haWwiLCJXVCI6Mn0%3D%7C0%7C%7C%7C&sdata=RIUm0FVceAN4fyhqLhvM2jwFCintFuIi8Wez%2BxIofBw%3D&reserved=0) requires authors to make all data underlying the findings described in their manuscript fully available without restriction, with rare exception (please refer to the Data Availability Statement in the manuscript PDF file). The data should be provided as part of the manuscript or its supporting information, or deposited to a public repository. For example, in addition to summary statistics, the data points behind means, medians and variance measures should be available. If there are restrictions on publicly sharing data—e.g. participant privacy or use of data from a third party—those must be specified.

Reviewer #1: Yes

Reviewer #2: Yes

4. Is the manuscript presented in an intelligible fashion and written in standard English? 
 
PLOS ONE does not copyedit accepted manuscripts, so the language in submitted articles must be clear, correct, and unambiguous. Any typographical or grammatical errors should be corrected at revision, so please note any specific errors here.

Reviewer #1: Yes

Reviewer #2: Yes

5. Review Comments to the Author 
 
Please use the space provided to explain your answers to the questions above. You may also include additional comments for the author, including concerns about dual publication, research ethics, or publication ethics. (Please upload your review as an attachment if it exceeds 20,000 characters)

**Response to reviewers**: We thank you for your time and putting your effort into reviewing our manuscript. We find the comments valuable and increasing the merits of our study. Below are our detailed responses to the comments.

Reviewer #1: The manuscript “Investigating the moments of “aha” and “hmm” through acoustic analysis of voice and speech in pre-service physics teacher education” presents interesting results when analyzing spontaneous speech during interaction of future teachers. Some issues have been cited in previous reviews. 
In my opinion, presenting 10 subjects in your analysis is not a demerit. Treating and analyzing spontaneous speech samples is laborious and generates a huge amount of data, presented adequately by the authors, in addition to being a common format in the field of experimental phonetics. However, the data cannot be generalized beyond the study sample and the authors should make this clearer in the discussion, in addition to highlighting the fact that it is valid when checking people wearing masks.

**Response**: We find this comment very useful. We have now added to the discussion: Lines 455-461: This present study is piloting new research methods for learning research, and for generalizing the results, other situations and environments for the learning discussions should be studied. Additionally, this study was conducted in Finnish cultural and language context. The results may be affected by the cultural use of voice and, thus, the characteristics of voice in other languages and cultural contexts should be investigated. Also, this study concentrated on peer discussions and teacher-student dialogues were not considered. Future research could investigate teacher´s influence on student´ vocal manifestations of the learning moments.

I suggest that in the summary it is clarified that the LTAS measurement is extracted instead of “voice quality through spectrum analysis”, it is clearer for the reader, as with this information alone it is not possible to understand what else is being measured.

**Response**: This is a good point. We have now replaced this as suggested, line 25: acoustic voice quality measured by LTAS

It is necessary to make it clear in the method what the authors are referring to when they mention “aha” and when they mention “hmm”. I understand what they are by deduction but in the section “Recognizing the “aha” and “hmm” moments” (page 15) the authors should make this clearer.

**Response**:

Thank you for pointing this out. We have now clarified this. Previous text was: From the recordings, samples indicating either confusion (category 1), understanding (2) or explaining (3) were extracted by one of the researchers based on context. The new and clearer description is now: Lines 242-246: From the recordings, samples indicating either confusion, that is when students expressed uncertainty or an impasse in resolving the task or conception (category 1), understanding, i.e. the moments where students expressed an insight of an idea or a conception (2), or explaining, the speech turns where students provided information to peer (3) were extracted by one of the researchers who based the categorization also on context of discussion.

Also, we would like to point out that the reliability of the classification was checked through interrater reliability measures, outlined on lines 254-258.

I congratulate the authors for the quality of the manuscript and for their work in adapting the revisions requested in previous rounds.

**Response**: We thank the reviewer for recognizing the merits of the study and appreciating the revisions made for improving the article.

Reviewer #2: This manuscript addresses an interesting aspect related to learning. From what I can understand, the authors addressed many of the concertns raised by the other reviewers. 
I suggest to add more detail to the general idea of Problem-based learning, that is cited only at the beginning of tyhje manuscript, with only a single literature reference.

**Response**: We appreciate this comment. We have added more details concerning problem-based learning.

Lines 42-44: In problem-based learning, the learners orient and analyse the problem, engage in self-directed problem solving, and end with a reporting activity such as explaining their answer to teachers or peers.

Line 48: Added a new reference [2], Yew EH, Goh K. Problem-based learning: An overview of its process and impact on learning. Health professions education. 2016;2(2):75-79. https://doi.org/10.1016/j.hpe.2016.01.004

Lines 226-235: We added problem-based learning when suitable: The teaching sessions were structured for problem-based learning in pairs or small groups. All the pairs were given the problem ”Rank the light bulbs in DC circuits in the order of their relative brightness” (Fig 2). During the first two recordings, tasks became more complex per each task. In the third recording, the students were given some of the same tasks as in first two recordings - in order to evaluate the progress of learning or understanding that took place. The directions for the problem-based sessions followed the Predict-Observe-Explain structure: First on pen and paper only –analysing the problem first individually and then discussing the prediction as a pair and possibly reaching a consensus – and then constructing the circuits and observing if their prediction was confirmed or if revisions were needed. Based on the observation, the pair formed their final explanation, which was reported to the teacher.

Lines 491-492: This will give information on emotional reactions in the learning process, for example at different stages of problem-solving or with differences in difficulty of the problems.

Moreover, the authors should add detail on how they found, in their analysis, the thre categories: confusion, understanding, and explaining. Just saying that they "were extracted by one of the researchers based on context" appears to me too vague. Detail on analysis methods used to extract them is necessary.

**Response**: We appreciate this comment. We have now strengthened the categorization of the samples. Previous text was: From the recordings, samples indicating either confusion (category 1), understanding (2) or explaining (3) were extracted by one of the researchers based on context. The new and clearer description is now, Lines 242-246: From the recordings, samples indicating either confusion, that is when students expressed uncertainty or an impasse in resolving the task or conception (category 1), understanding, i.e. the moments where students expressed an insight of an idea or a conception (2), or explaining, the speech turns where students provided information to peer (3) were extracted by one of the researchers who based the categorization also on context of discussion.

Also, we would like to point out that the reliability of the classification was checked through interrater reliability measures, outlined on lines 254-258.

6. PLOS authors have the option to publish the peer review history of their article ([what does this mean?](https://eur03.safelinks.protection.outlook.com/?url=https%3A%2F%2Fc05y1x9s.r.us-east-2.awstrack.me%2FL0%2Fhttps%3A%252F%252Fjournals.plos.org%252Fplosone%252Fs%252Feditorial-and-peer-review-process%2523loc-peer-review-history%2F1%2F010f0191948db704-7c83b35d-16b5-454c-8766-c1d23c62641e-000000%2Fi7Zx8tcSB2EPvpUYcsyYDLECe0I%3D174&data=05%7C02%7Ckathanja%40jyu.mail.onmicrosoft.com%7C7a07683149de4d13737608dcc6b0d93a%7Ce9662d58caa44bc1b138c8b1acab5a11%7C1%7C0%7C638603711603565893%7CUnknown%7CTWFpbGZsb3d8eyJWIjoiMC4wLjAwMDAiLCJQIjoiV2luMzIiLCJBTiI6Ik1haWwiLCJXVCI6Mn0%3D%7C0%7C%7C%7C&sdata=bnBAfI6LSQKopbQNRpXmo55yxCvp05%2B4xli8D7cevDo%3D&reserved=0)). If published, this will include your full peer review and any attached files. 
 
 
If you choose “no”, your identity will remain anonymous but your review may still be made public. 
 
 
**Do you want your identity to be public for this peer review?** For information about this choice, including consent withdrawal, please see our [Privacy Policy](https://eur03.safelinks.protection.outlook.com/?url=https%3A%2F%2Fc05y1x9s.r.us-east-2.awstrack.me%2FL0%2Fhttps%3A%252F%252Fwww.plos.org%252Fprivacy-policy%2F1%2F010f0191948db704-7c83b35d-16b5-454c-8766-c1d23c62641e-000000%2F9mUu0e5iPfqAl8w8BuMX_5YdsxE%3D174&data=05%7C02%7Ckathanja%40jyu.mail.onmicrosoft.com%7C7a07683149de4d13737608dcc6b0d93a%7Ce9662d58caa44bc1b138c8b1acab5a11%7C1%7C0%7C638603711603571842%7CUnknown%7CTWFpbGZsb3d8eyJWIjoiMC4wLjAwMDAiLCJQIjoiV2luMzIiLCJBTiI6Ik1haWwiLCJXVCI6Mn0%3D%7C0%7C%7C%7C&sdata=MAd5e2zMk%2BpCLPprR8ZvAHzBGckv8tHsuOU3lW%2BDEJM%3D&reserved=0).

Reviewer #1: No

Reviewer #2: No
